# Recurrent Cryptogenic Ulcerative Tracheobronchitis 6 Years After Lung Transplantation: Case Report

**DOI:** 10.1155/crit/5525333

**Published:** 2026-08-27

**Authors:** Samuel Brookes, Paroma Sarkar, Thomas Crowhurst, Phan Nguyen, Aeneas Yeo, Kate D. Lynch, Helen Whitford, Mark Holmes, Chien-Li Holmes-Liew

**Affiliations:** ^1^ Department of Thoracic Medicine, Royal Adelaide Hospital, Adelaide, South Australia, Australia, rah.sa.gov.au; ^2^ School of Medicine, University of Adelaide, Adelaide, South Australia, Australia, adelaide.edu.au; ^3^ Respiratory Unit, Lyell McEwin Hospital, South Australia, Australia, sahealth.sa.gov.au; ^4^ Department of Gastroenterology and Hepatology, Royal Adelaide Hospital, Adelaide, South Australia, Australia, rah.sa.gov.au; ^5^ Department of Respiratory Medicine, Townsville University Hospital, Townsville, Queensland, Australia

**Keywords:** bilateral sequential lung transplantation, case report, Crohn’s disease, infliximab, tracheobronchitis

## Abstract

**Background:**

Tracheobronchitis is an uncommon pattern of airway disease and is typically caused by infection, inhalation injury, infiltrative disorders and more rarely, systemic autoimmune disease. Rarer again is the development of bronchial webbing following tracheobronchitis. The underlying aetiology is usually identified through biopsy, culture or a detailed clinical history. We present a case of cryptogenic ulcerative tracheobronchitis with bronchial webbing being successfully managed by bilateral lung transplantation that has, however, been complicated by recurrence.

**Case Presentation:**

In 2019, Crowhurst et al. detailed the case of a patient who presented with dyspnoea and haemoptysis. Bronchoscopy revealed ulcerative tracheobronchitis and he subsequently formed recurrent web‐like stenoses of the central bronchi, leading to rapidly progressive type 2 respiratory failure; he received bilateral sequential lung transplantation for definitive management. At the time of transplant, the presumed diagnosis was an inhalation injury that occurred while travelling in Vietnam, or exposure to the organophosphate pesticide Malathion several days prior. The explanted lung histology revealed ulcerating bronchitis with intense histiocytic infiltrates, bronchial luminal fibrosis and obstruction with scant, poorly formed granulomas. The patient thrived with stable lung function and had minimal complications initially post‐transplantation. However, 6 years after lung transplantation and without re‐exposure, the patient re‐developed ulcerative tracheobronchitis, which shared similar histopathologic features to airway involvement of Crohn’s disease. Gastrointestinal or other organ system involvement of Crohn’s disease was not established despite extensive investigations. A decision for phenotypic treatment with the TNF‐*α* inhibitor, infliximab, was made with subsequent rapid improvement in the tracheobronchitis.

**Conclusion:**

This is the first case documented in the literature of ulcerative tracheobronchitis followed by intense bronchial web‐like stenoses caused by an unspecified autoimmune disease, treated by bilateral lung transplantation. The disease recurred after 6 years and responded to phenotypic management with the TNF‐*α* inhibitor, infliximab. The patient will require careful monitoring given the risk of augmented immunosuppression and unknown disease trajectory.

## 1. Background

Large airway diseases such as tracheobronchitis (TB) are uncommonly reported in the literature. They are generally caused by viral, bacterial or fungal infection, thermal or abrasive inhalation injury, infiltrative disorders such as amyloidosis and autoimmune diseases such as granulomatosis with polyangiitis (GPA) or inflammatory bowel disease (IBD), including Crohn’s disease (CD) and ulcerative colitis (UC). In the lung transplant cohort, airway disease can be a manifestation of acute and chronic lung allograft dysfunction. A report published by Crowhurst et al. in 2019 highlighted a rare case of ulcerative TB with the development of bronchial‐web like stenoses refractory to interventional bronchoscopy techniques that ultimately required bilateral sequential lung transplantation (BSLT) for definitive management [1]. Here, we present an update for the same patient, who re‐developed ulceration involving both native airways and the allograft. We hope to provide an experiential insight into the diagnostic challenges and the phenotypic treatment approach delivering early remission of the disease, delaying potential need for re‐transplant.

## 2. Case Presentation

**Figure 1:**
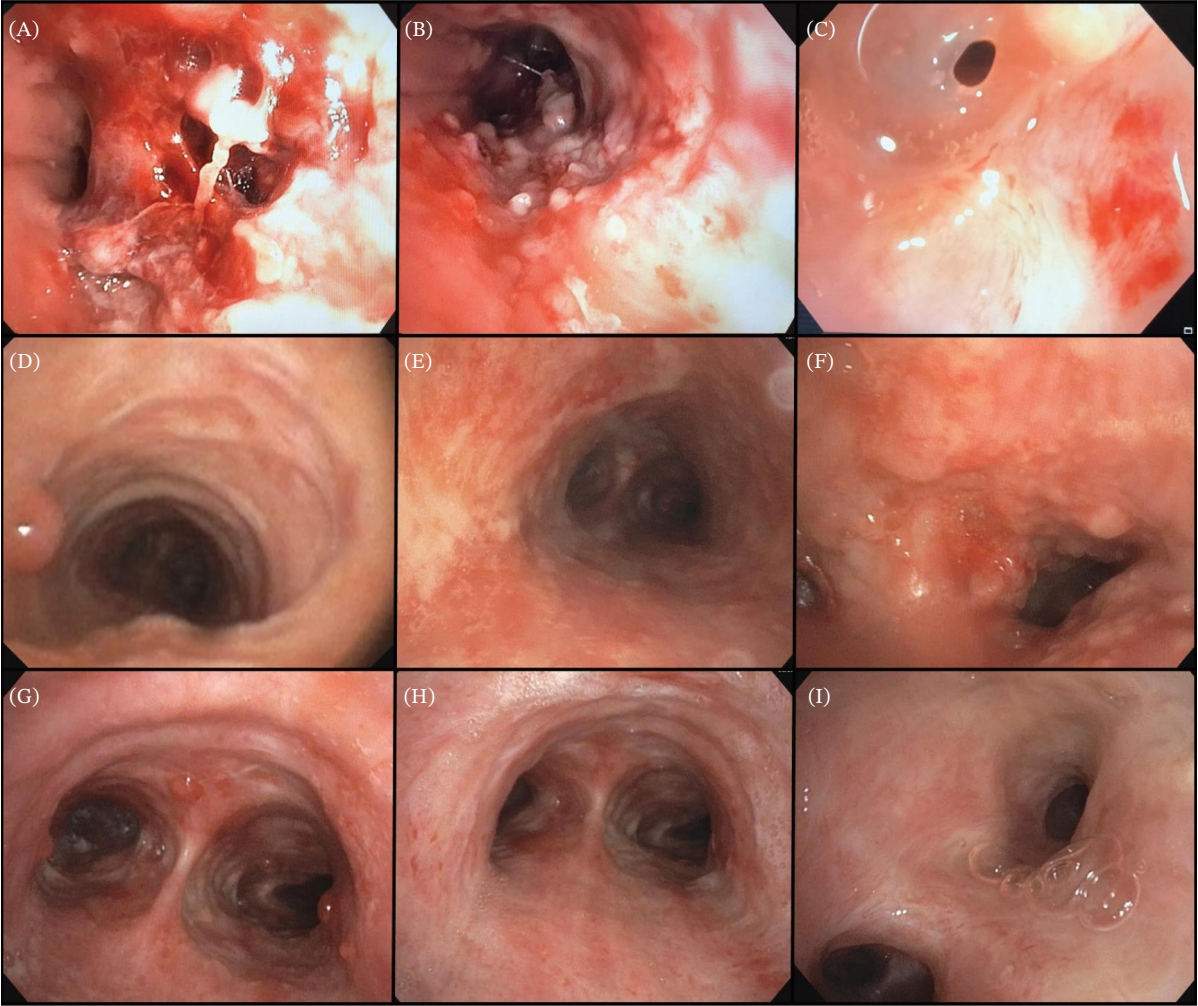
Serial bronchoscopy examination of large airways. (A–C) Pre‐transplant. (D–I) post‐transplant. (A) Severe bronchial ulceration with spontaneous bleeding. (B) Tracheal ulceration and nodularity. (C) Bronchial web‐like stenosis. (D) Proximal left‐sided tracheal nodule in 2019. (E–F) Mucosal ulceration and nodularity after re‐presenting in February 2024. (G) After two doses of infliximab with reduction in mucosal inflammation and few inflammatory and haemorrhagic appearing nodules in April 2024. (H–I) After four doses of infliximab with marked improvement in mucosal changes and resolution of nodularity.

**Figure 2:**
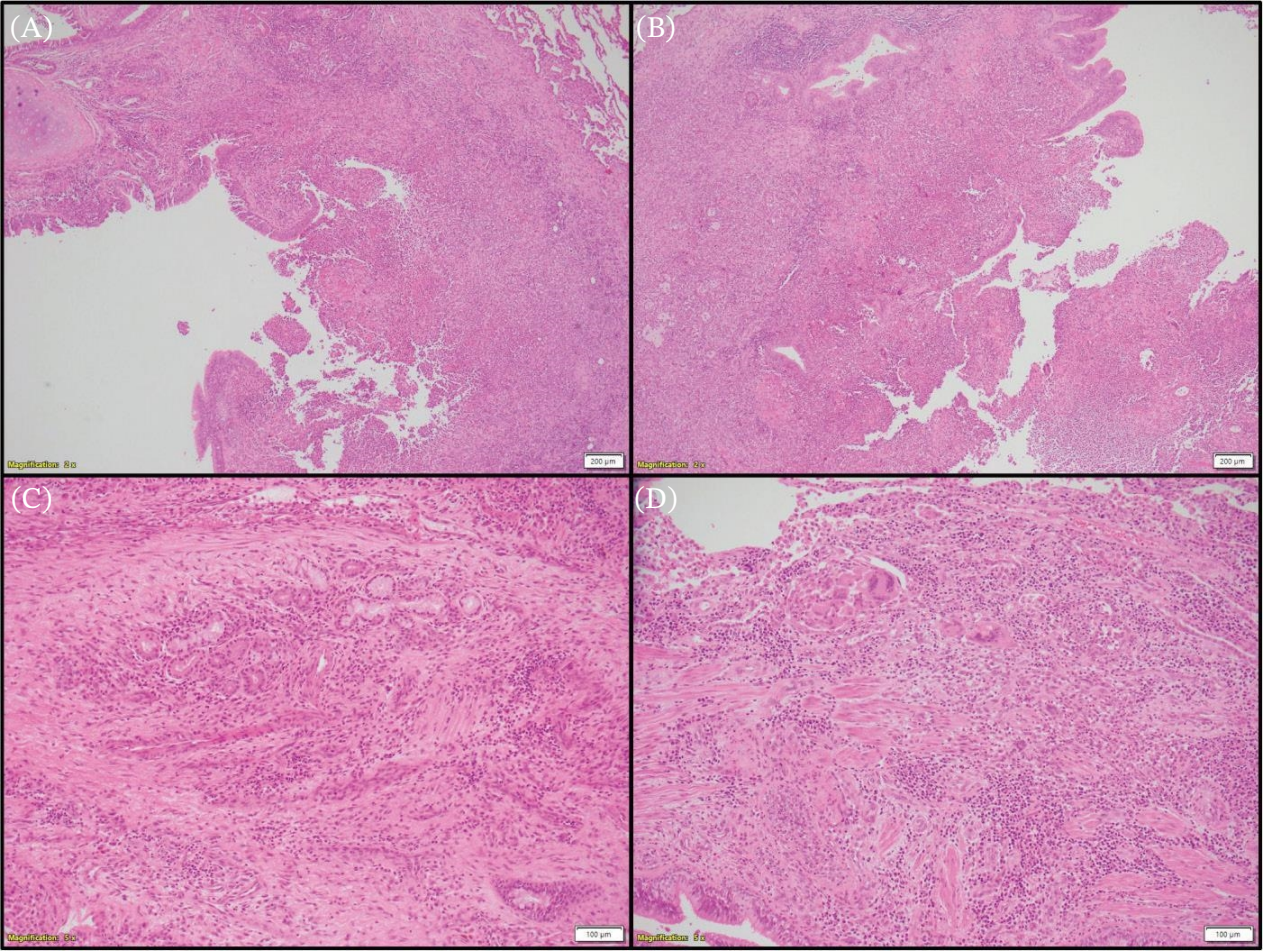
Explanted lung histology with haematoxylin and eosin staining. (A) Acute bronchitis with ulceration and chronic inflammation including giant cells. (B) Bronchial wall ulceration with suppuration with frequent giant cells. (C) Bronchitis with luminal obstruction and fibrosis. (D) Poorly formed non‐necrotising granuloma within the bronchial wall.

A 53‐year‐old man presents to the Royal Adelaide Hospital 6 years post BSLT in February 2024 with a 2‐week history of cough, haemoptysis and dyspnoea unresponsive to oral antibiotics and corticosteroids. He resides in rural South Australia and works as an electrician. Prior to transplantation, he had a 25 pack‐year history of cigarette smoking and had relapsed for 6 months after BSLT. He ceased 4 years prior to the current presentation. At the time of BSLT in May 2018, because of the rapidly progressive type 2 respiratory failure with ulcerative TB refractory bronchial web‐like stenoses (Figure 1A–C), the differential diagnoses were inhalation injury from exposure to Malathion, an organophosphate pesticide, an alternative inhalant during travel in Vietnam or an occult autoimmune condition. In the weeks prior to transplantation, the patient required periods of mechanical ventilation and venovenous extracorporeal membrane oxygenation and had numerous bronchoscopies including interventions such as web‐puncture using Wang needle followed by balloon dilatation of segmental bronchial webs. Histopathology of explanted lungs demonstrated bronchial ulceration, fibrotic bronchial luminal stenoses most pronounced in central bronchi, with rare and poorly formed granulomas (Figure 2A–D). There was no significant vasculitis. Overall, the explanted lungs resembled severe ulcerative bronchitis as can rarely be seen in diseases such as CD.

**Figure 3:**
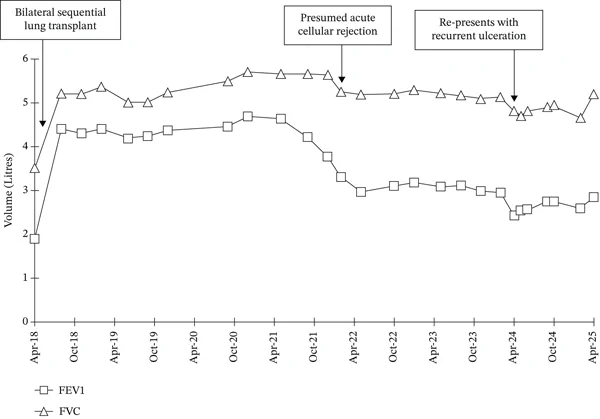
Timeline of major events and serial spirometry. Forced expiratory volume in 1 s (FEV1) and forced vital capacity (FVC) between April 2018 (pre‐transplant) and April 2025. First arrow (from left)—bilateral sequential lung transplant was performed in May 2018. Second arrow—presumed acute cellular rejection was treated with pulse methylprednisolone in November 2021, although FEV1 did not return to pre‐treatment baseline. Third arrow—presentation with dyspnoea and haemoptysis in February 2024 where further decline in FEV1 occurred.

His immediate post‐transplant course had been uncomplicated. In 2019, surveillance bronchoscopy revealed a proximal tracheal nodule (Figure 1D), which resolved shortly thereafter and was not biopsied. He had unremarkable routine transbronchial biopsies and stable pulmonary function tests until 2021, where a decline in spirometry prompted treatment for presumed lung allograft dysfunction with intravenous methylprednisolone, which led to stabilisation in spirometry (Figure 3). Bronchoscopy was unremarkable, broncho‐alveolar lavage cultures negative, transbronchial biopsies were ISHLT A0B0 and serum donor‐specific antibodies were negative. He was maintained with tacrolimus 4 mg BD, mycophenolate mofetil 1 g BD, prednisolone 5 mg and antimicrobial prophylaxis with trimethoprim/sulfamethoxazole. Other medications included azithromycin and sertraline.

**Figure 4:**
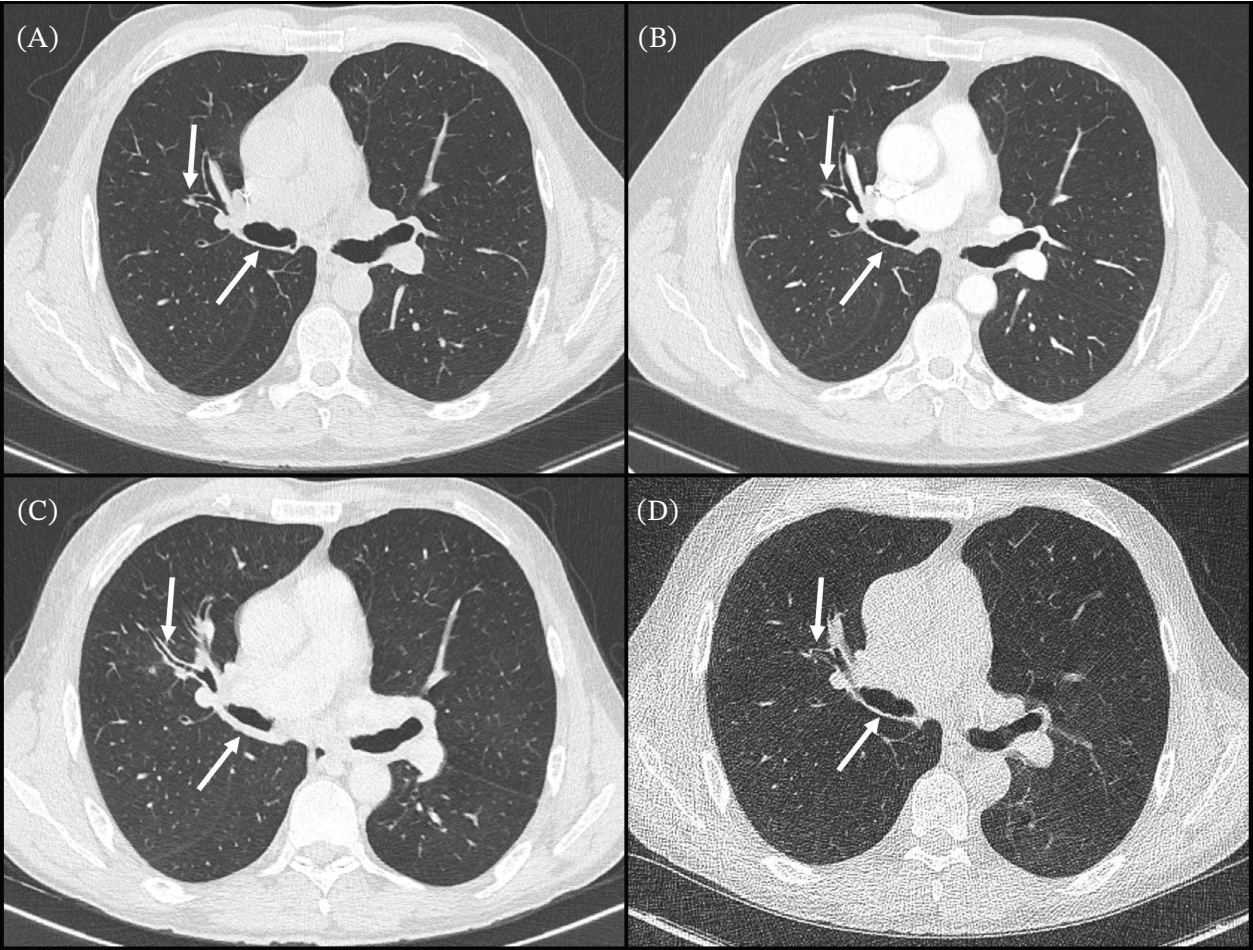
Axial and coronal CT imaging of the chest. (A) December 2018—7 months after transplant with normal bronchial wall diameter (see arrow). (B) July 2019—13 months after transplant with stable bronchial wall diameter. (C) February 2024—marked progression in bronchial wall thickening. (D) April 2025—improvement in bronchial wall thickening after 14 months of treatment with infliximab closer to pre‐treatment baseline.

On arrival to the emergency department at his current presentation, routine examination was unremarkable with vital signs within normal ranges. Blood samples revealed elevated C‐reactive peptide of 160 mg/L with elevated white cell count with neutrophilia of 21.0 × 10^9^/L. Sputum culture, blood culture and respiratory viruses were negative. Computed tomography (CT) chest was performed, which revealed left lower lobe consolidation and marked bronchial wall thickening, which was new since transplantation on serial CT imaging (Figure 4). He was commenced on piperacillin‐tazobactam and daily azithromycin. Bronchoscopy revealed mucosal inflammation, ulceration and nodularity in both native airways (trachea and proximal right and left bronchi) and allograft airways, extending to third generation bronchi (Figure 1E,F). Bronchial stenoses were not identified. Endobronchial biopsies revealed similar histopathologic changes compared with pre‐transplant with histiocytic infiltration, mucosal ulceration and fibrinoid necrosis. Biopsies were sent for culture and human papilloma virus (HPV) testing, which were negative. No features of vasculitis were identified acknowledging the superficial biopsy location. Bronchoalveolar lavage was culture negative with low‐titre cytomegalovirus detected (1585 copies/millilitre), which was not thought to be contributing to the presentation. Piperacillin‐tazobactam was continued and prednisolone dose increased from 5 mg to 25 mg daily. Upon questioning, the patient reported no occupational or inhalational exposures and specifically, no Malathion exposure or travel within months. Repeat blood samples to investigate autoimmune and infective processes were performed including: antinuclear antibody, extractable antigen nuclear test, rheumatoid factor, anti‐citrullinated protein antibody, antineutrophil cytoplasmic antibodies, anti‐glomerular basement membrane antibody, anti‐double stranded deoxyribonucleic acid antibody, human immunodeficiency virus, hepatitis B virus, hepatitis C virus and interferon gamma release assay for tuberculosis. These were all negative.

The diagnosis was relapse of an unspecifiable autoimmune disease responsible for the pre‐transplant airway ulceration. Given the explanted lung tissue shared similarities to ulcerative bronchitis of IBD, gastroenterology specialist advice was sought. Faecal calprotectin was within normal ranges (20 *μ*g/g), gastroduodenoscopy and ileocolonoscopy showed no definitive diagnosis of IBD with a single 3‐mm inflammatory polyp identified in the caecum that was removed with cold snare. Random biopsies of gastric and colonic mucosa revealed no microscopic evidence of IBD. CT abdomen/pelvis did not identify small bowel CD nor were there features of extrapulmonary disease suggestive of an alternate autoimmune process. There was no evidence of iron deficiency and as such, capsule endoscopy was not performed. The consensus multi‐disciplinary decision was to trial phenotypic management and the patient was commenced on infliximab. A 300‐mg intravenous loading began in February 2024 with 0, 2 and 6‐week infusions followed by infusions every 8 weeks thereafter. Serial bronchoscopy and spirometry were performed for treatment monitoring (Figure 1G–I, Figure 3) with improvement in both mucosal appearances and spirometry. The patient reports excellent symptom control after 1 year of infliximab therapy, describes no recurrence of haemoptysis, wheeze or dyspnoea and has not experienced any adverse or unexpected effects.

## 3. Discussion and Conclusions

We describe recurrence of ulcerating TB 6 years after BSLT. The disease prior to transplant was progressive, medically refractive and considered secondary to inhalation injury, noting exposure to the organophosphate pesticide Malathion several days prior. At this time, biopsies showed intense histiocytic infiltration of the bronchial wall, followed by luminal fibrosis and rapidly progressive bronchial webbing involving central bronchi. There are reports of inhalation injuries presenting similarly such as those caused by thermal injury and hydrochloric acid [2, 3]. At the time of transplantation, there was concern about the choice of anastomosis (tracheal or bilateral bronchial) due to risk of breakdown from ulceration and necrosis. Serial bronchoscopies had shown reduction in the degree of tracheal and proximal bronchial inflammation, so it was decided to proceed with bilateral bronchial anastomosis. Fortunately, despite recurrence of the disease, the anastomoses have remained intact and healthy. There are numerous reasons that inhalation injury is no longer felt likely. First, there was no repeat exposure to Malathion nor other inhalants prior to the recurrence. Second, the progression post‐transplant was less rapid than prior to transplant. Third, he did not develop the bronchial web‐like stenoses seen prior, suggesting an altered clinical course. Fourth, there are no other reports of Malathion causing this type of airway disease [4]. Fifth, the compelling response to phenotypic therapy with infliximab would render inhalation injury less likely.

The most likely diagnosis is of a cryptogenic systemic autoimmune condition. There is a wide array of causes of TB, most of which are readily diagnosed with routine biopsy and culture. Infection is a common cause of TB including *Candida* spp. and *Aspergillus* spp. in the immunosuppressed and lung transplant cohort [5]. Serial biopsy and bronchial washings were culture, stain and polymerase chain reaction negative and opportunistic infections by organisms such as HPV, tuberculosis and herpetic viruses were excluded. Advanced molecular pathogen detection such as next‐generation sequencing or 16S rRNA sequencing was not employed back in 2018 and as such, occult infection remains a possibility. The sustained response to additive immunosuppression with infliximab however renders this unlikely. Other causes include infiltrative disorders such as amyloidosis, which were readily excluded histologically. There was no history of airway trauma, surgery or radiotherapy. Congenital disorders such as tracheobronchopathia osteochondroplastica do not present in this fashion as they typically involve the cartilaginous airways, contrary to the circumferential disease seen in this case [6]. They also lack the degree of inflammation, necrosis and ulceration. Autoimmune disorders, particularly relapsing polychondritis (RP), sarcoidosis, GPA and IBD can be challenging to diagnose. RP typically presents with multi‐organ involvement, which was not seen, and although isolated airway involvement has been reported, it is readily steroid responsive [7, 8]. There was also involvement of the non‐cartilaginous airway on explant. In ANCA‐negative GPA, there are typical features histologically such as well‐formed granulomas, widespread necrosis and blood vessel wall infiltrates seen on adequate tissue sample [9, 10]. The absence of well‐formed granulomas and vasculitis on thorough examination of bilateral lung explant effectively excluded GPA, and serial ANCA was negative. There have been rare reports of tracheal and bronchial Pulmonary Langerhans Cell Histiocytosis (PLCH); however, these have been more associated with nodular or mass‐like obstruction rather than webbing. In our case, there were also no definitive features of PLCH such as the presence of Langerhans cells on histology, cyst formation or parenchymal nodular infiltrates [11–13]. Sarcoidosis was also considered as a differential; however, the pattern of disease, web formation, intense ulceration, absence of significant hilar or mediastinal lymphadenopathy and lack of well‐formed granulomas went against the diagnosis. Tracheobronchial involvement in sarcoidosis is usually milder, with a cobblestone appearance and can cause bronchial stenoses rather than webbing [14–16].

The diagnosis of IBD was unable to be definitively made given the paucity of gastrointestinal involvement. Thorough history did not reveal any symptoms suggestive of indolent or intermittent IBD. The patient underwent numerous gastroduodenoscopies and ileocolonoscopies since 2018 and these were serially negative, other than the previously mentioned isolated inflammatory polyp in 2024. A normal faecal calprotectin made subclinical IBD less likely and multiple CT abdomen/pelvis showed no suggestive features. In those with established Crohn’s, the literature suggests asymptomatic bronchial wall thickening can be seen in up to half on CT imaging [17]. There are case reports of TB in CD, but all had an established diagnosis, or the diagnosis was made concurrently at the time of presentation with airway disease [18–20]. Patients with severe airway disease of Crohn’s typically responded to higher doses corticosteroids, and one would anticipate that intravenous methylprednisolone and maintenance with antirejection medications with tacrolimus, mycophenolate and prednisolone would be sufficient to suppress the disease, which did not occur in this case [17–20].

The decision to treat with infliximab followed a multidisciplinary discussion taking into consideration the clinical, histopathologic and radiologic features. The decision for augmented immunosuppression above that of standard anti‐rejection medications is significant given the risks associated including infection and malignancy. This case adds to the literature that TNF‐*α* inhibitors appear to be safe, at least in short‐term, in solid organ transplant recipients [21]. There is a scarcity of data in the literature regarding both the short and long‐term safety of TNF‐*α* inhibitors in the lung transplant cohort. A case published by Abellan et al. in 2023 reported a good short‐term outcome following the use of both infliximab and tocilizumab in an individual with acute fibrinous organising pneumonia [22]. A consensus statement by the Journal of Heart and Lung Transplantation in 2021 highlights the absence of data in lung transplant recipients with all TNF‐*α* inhibitors [23]. Given the histopathologic similarities to airway involvement in those with established IBD, it was hypothesised that a shared pathway involving TNF‐*α* may be involved in the pathogenesis. A case report published by Horgan et al. in 2019 demonstrated a good clinical response to infliximab in a patient with UC related TB, further supporting its use in this case [24]. The positive response to infliximab reinforces the trial of phenotypic therapy and could be considered more broadly in those with similar histopathologic features. The decision to proceed with lung transplantation in this case was potentially controversial given the paucity of experience in the literature; however, given this patient is approaching the published median overall survival from lung transplantation of 7 years and has stabilised with therapy, this outcome could be considered a success in that context. With such a profound response to therapy, one must question whether treatment with TNF‐*α* inhibitors in 2018 could have prevented the need for lung transplantation, acknowledging the biopsy specimens of the time were less definitive until the explanted lungs were examined. Careful surveillance of lung function and serial bronchoscopies has been employed to ensure a sustained treatment response.

In conclusion, we report a unique case of ulcerative TB, which is presumed to be secondary to a cryptogenic autoimmune disease with similar features to airway CD although there was no identifiable gastrointestinal disease to confirm this diagnosis. The patient underwent urgent bilateral lung transplantation in 2018 and despite careful review of explanted lung histopathology and extensive investigations, a clear aetiology was not identified. Six years after transplant, the disease recurred in the native airways and allograft and has been successfully managed with the TNF‐*α* inhibitor, infliximab. The positive treatment response re‐enforces the empiric use of TNF‐*α* inhibitors to treat the disease phenotype and provides short‐term safety data for infliximab use in the lung transplant cohort. Close monitoring with lung function and direct visualisation by flexible bronchoscopy has been employed given the uncertainty of the disease process. This case highlights multiple challenges including the diagnosis of autoimmune ulcerative TB without multi‐organ involvement or diagnostic histopathology, the decision to perform urgent lung transplantation despite an uncertain disease process, the use of infliximab targeting histopathology similar to airway IBD in the absence of gastrointestinal involvement and the risks of compound immunosuppression in an already heavily immunocompromised patient.

**Nomenclature**
BSLT: bilateral sequential lung transplant
CD: Crohn’s disease
CT: computed tomography
GPA: granulomatosis with polyangiitis
HPV: human papilloma virus
IBD: inflammatory bowel disease
RP: relapsing polychondritis
TB: tracheobronchitis
UC: ulcerative colitis

## Author Contributions

Samuel Brookes wrote the main manuscript text, performed literature review and curated data.

Thomas Crowhurst authored the original manuscript and provided clinical insights, edited the manuscript.

Phan Nguyen and Aeneas Yeo edited the manuscript and assisted with general respiratory perspective, intervention and physiology perspectives.

Helen Whitford, Mark Holmes, Paroma Sarkar edited the manuscript and provided input from lung transplant perspective.

Kate D. Lynch edited the manuscript and provided input from gastroenterology and IBD perspective.

Chien‐Li Holmes‐Liew heavily involved in editing the manuscript and provided input from general respiratory, lung transplant and clinical overview.

## Funding

No funding was received for this manuscript.

## Ethics Statement

Ethics approval was not required for this case report. The patient provided informed written consent prior to commencement of writing.

## Consent

The authors declare that appropriate written informed consent was obtained for the publication of this manuscript and accompanying images.

## Conflicts of Interest

The authors declare no conflicts of interest.

## Data Availability

The data that support the findings of this study are available on request from the corresponding author. The data are not publicly available due to privacy or ethical restrictions.
